# B-Cell Immune Responses in Myocarditis: Mechanisms, B-Cell Receptor Repertoire, and Clinical Translation

**DOI:** 10.3390/cells15191734

**Published:** 2026-09-23

**Authors:** Xixiong Lin, Xinsheng Yao, Jun Li

**Affiliations:** Department of Immunology, Center of Immunomolecular Engineering, Zunyi Medical University, Zunyi 563003, China; lxx9876@zmu.edu.cn (X.L.);

**Keywords:** myocarditis, B cells, BCR repertoire, single-cell sequencing, autoantibodies

## Abstract

Myocarditis is an inflammatory myocardial disease induced by infection, autoimmunity, or immune checkpoint inhibitor (ICI) therapy. Its immunopathogenesis has long been attributed primarily to T-cell-mediated cellular immunity. Increasing evidence now indicates that B cells contribute to the initiation, persistence, and myocardial remodeling of myocarditis not only as a source of autoantibodies, but also through antigen presentation, cytokine secretion, and immunoregulatory crosstalk. The emergence of single-cell sequencing and B-cell receptor (BCR) repertoire sequencing has begun to reveal B-cell clonal expansion, immunoglobulin class switching, and dynamic shifts in functional subsets, offering a new window into humoral immune responses in myocarditis. This review synthesizes current evidence on BCR repertoire remodeling, autoantibody-mediated myocardial injury, functional heterogeneity among B-cell subsets, and the clinical translation of these findings. B cells can mediate myocardial damage through autoantibodies against adenine nucleotide translocator (ANT), cardiac myosin, and acetylcholine receptor (AChR), and their pathogenic effects depend on immunoregulatory networks such as Th17/IL-17 signaling and autophagy. Notably, B cells are functionally heterogeneous, TNF-α^+^ B cells promote persistent inflammation and myocardial fibrosis, whereas IL-10^+^ regulatory B cells (Bregs) confer immune protection by suppressing Th17 responses and maintaining Treg homeostasis. Studies of ICI-associated myocarditis suggest that BCR repertoire features and autoantibody profiles may provide insights into disease-associated immune remodeling. However, the clinical utility of these features as biomarkers remains uncertain, given the lack of standardized assays and sufficiently validated sensitivity, specificity, and reproducibility. In the future, integrating BCR repertoire, multi-omics, and functional subset analyses may improve characterization of disease-associated humoral immune responses and help identify candidate biomarkers for further clinical investigation.

## 1. Introduction

Myocarditis is an inflammatory myocardial disease induced by multiple factors, including viral infection, autoimmune dysregulation, and immune checkpoint inhibitors (ICIs) [1,2,3,4]. It can cause cardiomyocyte injury, cardiac dysfunction, malignant arrhythmias, and even sudden cardiac death, and it remains one of the leading causes of heart failure and dilated cardiomyopathy (DCM) in children and young adults [1,5,6,7]. Although the inflammatory responses triggered by different etiologies differ, sustained uncontrolled immune responses are considered a key determinant of disease severity and long-term prognosis [8,9,10]. Therefore, a deeper understanding of the immunopathogenesis of myocarditis is essential for precision diagnosis and targeted therapy.

For a long time, research on myocarditis has focused mainly on T-cell-mediated cellular immunity. Extensive evidence has confirmed that effector T-cell subsets, including CD4^+^ T cells, CD8^+^ T cells, and Th1/Th17 cells, can mediate myocardial injury through direct cytotoxicity or the release of proinflammatory cytokines, and contribute to disease progression toward chronic cardiomyopathy [11,12,13,14,15]. However, a T-cell-centered view alone cannot fully explain the complex immunopathological processes of myocarditis. As an important component of adaptive immunity, B cells not only differentiate into plasma cells that produce autoantibodies, but also participate in inflammatory responses through antigen presentation, cytokine secretion, and regulation of T-cell immune responses, playing a critical role in the initiation, persistence, and myocardial remodeling of myocarditis [16,17,18,19]. This is particularly evident in viral myocarditis [20,21], autoimmune myocarditis [22,23], and ICI-associated myocarditis [24,25], where B-cell-mediated humoral immune abnormalities have become a growing focus of research.

In recent years, the development of single-cell sequencing and B-cell receptor (BCR) repertoire sequencing has provided powerful tools for systematic analysis of B-cell immune responses in myocarditis. Existing evidence indicates that myocarditis is accompanied by marked BCR clonal expansion [26], immunoglobulin class switching [27], and reshaping of B-cell subsets [28], suggesting that antigen-driven humoral immune responses occur throughout multiple stages of disease onset and progression. Meanwhile, different functional B-cell subsets exhibit pronounced heterogeneity: pathogenic B cells can aggravate myocardial injury by producing autoantibodies and promoting inflammatory responses and tissue fibrosis [29,30], whereas regulatory B cells (Bregs) restrict excessive immune responses through mechanisms such as IL-10 secretion and maintenance of Treg homeostasis [21,31,32]. However, the functional division of labor among B-cell subsets, the dynamic evolution of antigen-specific BCRs, and the coordinated regulation between humoral and cellular immunity remain incompletely characterized, and their clinical translation value has yet to be fully defined. This review therefore focuses on the B-cell-mediated immunoregulatory network, synthesizes progress in BCR repertoire remodeling, autoantibody-mediated myocardial injury, functional heterogeneity of B-cell subsets, and clinical translation, and integrates recent evidence from single-cell omics and immune repertoire studies to discuss the potential of B-cell biomarkers in disease classification, risk assessment, and precision immunotherapy, with the aim of deepening our understanding of myocarditis immunopathogenesis and providing a basis for novel targeted therapeutic strategies.

To provide a comprehensive overview of the current understanding of B-cell involvement in myocarditis, we conducted a narrative literature search of PubMed, Web of Science, and Google Scholar for publications available through March 2026. The search used combinations of terms including “myocarditis,” “B cells,” “B-cell receptor,” “BCR repertoire,” “autoantibodies,” “regulatory B cells,” “viral myocarditis,” “autoimmune myocarditis,” and “immune checkpoint inhibitor-associated myocarditis.” Reference lists of relevant articles were also screened to identify additional studies. The literature was selected based on its relevance to B-cell-mediated mechanisms, BCR repertoire alterations, autoantibody responses, B-cell subsets, and their potential clinical and therapeutic implications in myocarditis. Both human and experimental studies were considered, with findings interpreted according to the corresponding study context.

## 2. BCR Repertoire Features in Myocarditis

B and T lymphocytes recognize specific antigens through unique adaptive immune receptors (AIRs) generated by somatic recombination. AIRs are produced through V(D)J recombination of gene segments and random somatic mutations, creating enormous receptor repertoire diversity [33,34]. Because each AIR is clonally expressed on individual cells, it determines the antigen specificity of that clone. Recent advances in bulk and single-cell sequencing, together with improved biostatistical modeling tools, have enabled in-depth characterization of AIR repertoires under various cardiac disease conditions [35,36]. Studies have shown that TCR repertoire analysis can help elucidate the pathophysiological mechanisms of cardiac diseases and may provide biomarkers for patient risk stratification, prognosis assessment, and identification of novel immunotherapeutic targets, although challenges such as interindividual HLA diversity and TCR cross-reactivity must be overcome [37]. By contrast, the role of BCR repertoires in cardiac diseases remains insufficiently studied and systematically characterized, leaving a clear research gap for future investigation.

Existing studies have shown that B cells undergo marked clonal remodeling during myocarditis. Single-cell BCR repertoire analysis has revealed significant clonal expansion of peripheral blood B cells during the acute phase of fulminant myocarditis in children, accompanied by biased usage of *IGHV* and *IGLV* genes and an increased proportion of IgM. As the disease enters the recovery phase, B cells progressively undergo immunoglobulin class switching from IgM to IgG and IgA, suggesting that the acute phase is dominated by initial humoral immune responses, whereas the recovery phase gradually establishes mature antigen-specific immune memory [38]. This dynamic pattern further indicates that B-cell responses are not a static process, but undergo continuous immune remodeling as the disease evolves.

In addition to clonal expansion, different functional B-cell subsets also show marked dynamic changes. Studies have found that regulatory B cells (Bregs) maintain low expression of immunosuppression-related genes, including *TGFB1* and *TGFBR2*, during both the acute and recovery phases [38]. This suggests that their immunoregulatory function may be chronically impaired, thereby weakening the negative regulation of excessive inflammatory responses. This finding suggests that, although the overall level of inflammation decreases during the recovery phase of myocarditis, immune homeostasis has not been fully restored, and persistent Breg dysfunction may represent an important immunological basis for chronic inflammation and myocardial remodeling.

Similar BCR remodeling has also been observed in ICI-associated myocarditis. Single-cell BCR sequencing revealed significant BCR clonal expansion during disease onset, with marked changes in V/J gene usage profiles in the CDR3 region compared with the recovery phase [26]. Because the CDR3 region determines BCR antigen recognition specificity, these changes suggest that ICI-associated myocarditis also involves a persistent antigen-driven B-cell response, possibly accompanied by recognition of new autoantigens. In addition, a recent case study of fulminant myocarditis combining scRNA-seq, CITE-seq, and TCR/BCR-seq analysis found pronounced immune activation during the acute phase. After JAK inhibitor treatment, the patient’s TCR/BCR repertoires gradually returned toward a near-physiological homeostatic state, further suggesting that dynamic changes in adaptive immune receptor repertoires are closely related to disease activity and clinical outcomes [39].

Overall, research on BCR repertoires in myocarditis is still in its early stage. Nevertheless, existing studies have observed BCR clonal expansion, biased V(D)J gene usage, immunoglobulin class switching, and restoration of BCR homeostasis during disease recovery in children with fulminant myocarditis, in patients with ICI-associated myocarditis, and in a case study of fulminant myocarditis [26,38,39]. Together, these findings indicate that BCR repertoire remodeling accompanies changes in humoral immune responses during myocarditis. However, the current evidence is derived from limited clinical cohorts, specific myocarditis subtypes, and individual case observations, and therefore does not yet establish whether particular BCR repertoire features are reproducibly associated with disease classification, treatment response, or prognosis. Future longitudinal studies integrating single-cell transcriptomics, spatial transcriptomics, and clinical phenotypes are needed to determine whether BCR repertoire features can serve as robust biomarkers and to assess their potential clinical utility in myocarditis.

## 3. B-Cell-Mediated Autoimmune Injury in Myocarditis

Autoimmune responses are a major driver of the initiation, persistence, and progression of myocarditis to dilated cardiomyopathy (DCM) [19,40]. For a long time, research has focused mainly on T-cell-mediated cellular immune injury. However, growing evidence indicates that B cells play a key role in the immunopathogenesis of myocarditis (Figure 1). Through multiple mechanisms, including autoantibody production, antigen presentation, and interaction with effector T cells, they contribute to the establishment of a persistently amplified autoimmune network [3].

### 3.1. Autoantibodies Are Core Effector Molecules Mediating B-Cell-Induced Myocardial Injury

There is currently broad consensus that autoantibodies are the most direct effector molecules through which B cells mediate myocardial injury. Early studies found that peripheral blood B cells transformed by Epstein–Barr virus (EBV) can produce human monoclonal antibodies that recognize cardiac myosin heavy chain, indicating that B cells have the potential to generate cardiac autoantibodies. Once the integrity of the cardiomyocyte membrane is compromised, these antibodies can enter cells and bind their corresponding antigens, thereby contributing to myocardial injury [41]. Subsequent animal experiments further confirmed the direct pathogenic effects of autoantibodies. After transplantation of peripheral blood leukocytes from patients with myocarditis into severe combined immunodeficiency (SCID) mice, autoantibodies against adenine nucleotide translocator (ANT) were detected in the recipient animals, accompanied by markedly reduced left ventricular systolic function [42,43]. Further mechanistic studies showed that anti-ANT autoantibodies can interfere with mitochondrial ATP/ADP transport, leading to impaired energy metabolism in cardiomyocytes and directly weakening myocardial contractility [44]. In addition to ANT, α-myosin heavy chain (α-MyHC) is among the best-characterized cardiac autoantigens. SEREX screening revealed that B-cell immune responses in experimental autoimmune myocarditis are highly focused on α-MyHC, accompanied by pronounced epitope spreading, whereas cardiotropic viral infection can also induce autoantibody responses against this antigen [16]. Together, these findings indicate that B-cell-derived autoantibodies are not only important mediators of myocardial injury but may also drive the gradual expansion of immune responses from viral antigens to cardiac self-antigens, accelerating the progression toward chronic cardiomyopathy.

### 3.2. The Th17/IL-17 Axis Continuously Amplifies B-Cell Autoimmune Responses

The sustained production of B-cell autoantibodies is not an isolated event; rather, it depends on T-cell help to form a persistently amplified immune loop. Among the relevant regulators, Th17 cells and their secreted IL-17 are considered key factors promoting B-cell activation. Clinical evidence indicates that the proportion of peripheral blood Th17 cells is markedly increased in patients with acute viral myocarditis and positively correlated with serum levels of anti-ANT autoantibodies [45]. Mechanistic evidence from experimental models further supports this association. In a CVB3-induced mouse model, neutralization of IL-17 reduced anti-ANT autoantibody production, while in vitro blockade of IL-17 signaling suppressed CD19^+^ B-cell proliferation and antibody secretion [46].

In addition to promoting B-cell proliferation, IL-17 regulates plasma cell differentiation. Studies in experimental autoimmune myocarditis have shown that IL-17 induces autophagy activation in B cells, increases *LC3B* expression, and upregulates *Blimp*-*1*, a key transcription factor for plasma cell differentiation, thereby promoting B-cell differentiation into CD38^+^CD138^+^ plasma cells. Conversely, the autophagy inhibitor 3-methyladenine (3-MA) markedly suppressed these processes and reduced anti-α-MyHC autoantibody levels [22]. Therefore, the Th17/IL-17 axis not only promotes B-cell activation but also facilitates plasma cell formation through the regulation of autophagy, thereby sustaining persistent humoral immune responses.

### 3.3. ICI-Associated Myocarditis Provides Further Evidence for B-Cell-Mediated Autoimmune Mechanisms

In recent years, ICI-associated myocarditis has provided new clinical evidence for understanding B-cell-mediated autoimmune mechanisms. Proteomic array analysis showed that anti-*RBPJ* autoantibodies remained persistently positive before treatment and during disease onset in patients with ICI-associated myocarditis, whereas anti-*EIF4EBP3* autoantibodies increased markedly over the course of the disease [24]. Further pathway enrichment analysis revealed that the target proteins of these autoantibodies are mainly involved in immune-related pathways, including B-cell receptor signaling and leukocyte transendothelial migration, and are closely associated with autoimmune diseases such as dilated cardiomyopathy and myasthenia gravis [24]. In addition, clinical case studies found that patients positive for anti-acetylcholine receptor (AChR) autoantibodies are more likely to develop severe ICI-associated myocarditis, with autoantibody levels significantly correlated with disease severity [47]. Collectively, these clinical observations indicate that humoral immune abnormalities are associated with myocarditis across different etiologies. However, whether specific autoantibodies can serve as reliable biomarkers for disease risk stratification or therapeutic response monitoring requires validation in larger, prospective clinical cohorts.

In summary, B-cell-mediated autoimmune injury is an important mechanism underlying the persistent progression of myocarditis. Its core features include autoantibody production, Th17/IL-17-dependent immune amplification, and sustained plasma cell differentiation. However, the contribution of B cells may differ according to the etiology of myocarditis. In viral myocarditis, B-cell responses may initially contribute to antiviral humoral immunity, whereas persistent immune activation and expansion of cardiac autoantigens may subsequently promote autoreactive antibody responses and chronic myocardial injury [9,17,20]. In autoimmune myocarditis, B-cell activation is more directly associated with autoreactive humoral responses and may interact with Th17/IL-17-mediated inflammation to amplify myocardial injury [22,46]. In ICI-associated myocarditis, immune checkpoint disruption may break immune tolerance and promote autoreactive immune responses [2]; although alterations in BCR repertoires, autoantibody profiles, and B-cell-related factors such as *CXCL13* have been reported [24,26], the extent to which B cells directly contribute to disease pathogenesis remains to be determined. Thus, although B-cell dysregulation is observed across different forms of myocarditis, its functional role and relative contribution may vary according to disease etiology and immunological context. In the future, systematic analysis of autoantibody repertoire evolution and the corresponding pathogenic B-cell clones may not only clarify the immunological basis of myocarditis chronification, but also identify candidate biomarkers and therapeutic targets based on autoantibody profiles and BCR repertoires.

## 4. Functional Heterogeneity of B-Cell Subsets in Myocarditis

In recent years, extensive studies have shown that B cells are not a functionally homogeneous population, but are composed of multiple subsets with distinct immunological functions, and their roles in myocarditis exhibit pronounced spatiotemporal heterogeneity (Figure 2). Different B-cell subsets can participate in disease onset and progression through proinflammatory or immunoregulatory mechanisms, and the balance between these functions determines not only the intensity of inflammatory responses, but also cardiac injury repair and disease outcomes [46,48]. Therefore, a detailed understanding of the functional characteristics and dynamic changes in different B-cell subsets is essential for deciphering the immunopathogenesis of myocarditis and developing precision immunotherapy.

### 4.1. Pro-Inflammatory B Cells Drive Sustained Myocardial Inflammation and Remodeling

Existing studies have shown that pro-inflammatory B cells are important effector cells driving persistent inflammation and tissue remodeling in myocarditis. Among them, TNF-α^+^ B cells can promote myocardial fibrosis and deterioration of cardiac function by secreting TNF-α and other inflammatory cytokines. Their proportion is negatively correlated with left ventricular ejection fraction (LVEF) and positively correlated with type III procollagen (PCIII) levels [18]. In addition, B cells can further amplify local inflammatory responses by inhibiting M2 macrophage polarization [49], promoting Th1 and Th17 cell differentiation, and suppressing Th2 immune responses [50]. In TNF-α-overexpressing mice, B-cell-mediated autoantibody deposition is markedly increased and eventually induces cardiac decompensation [51], whereas B-cell-deficient mice show significantly reduced inflammatory infiltration and myocardial injury [49], supporting a potential pathogenic role of pro-inflammatory B cells in disease progression.

Notably, pro-inflammatory B cells and Th17 cells form a persistently amplified positive-feedback regulatory network. Th17 cells can promote B-cell production of cardiac autoantibodies [45], and IL-17 can induce B-cell autophagy and plasma cell differentiation, thereby further enhancing autoantibody generation [22]. This persistently amplified immune loop not only maintains inflammatory responses, but may also promote the progression from acute myocarditis to chronic cardiomyopathy.

### 4.2. Regulatory B Cells Maintain Immune Tolerance and Limit Inflammatory Injury

In contrast to pro-inflammatory B cells, regulatory B cells (Bregs) are an important negative regulatory population that maintains immune homeostasis, mainly by secreting immunosuppressive cytokines such as IL-10 and TGF-β to limit excessive inflammatory responses [48,52]. In viral myocarditis models, PGE_2_ promotes the expansion of IL-10^+^ B10 cells through MAPK/AKT-AP1 and AhR signaling [28]. Adoptive transfer of B10 cells reduces the proportions of Th1 and Th17 cells, downregulates *T*-*bet* and *RORγt* expression, and significantly prolongs survival in experimental animals [21]. In contrast, clinical studies in patients with dilated cardiomyopathy have shown reduced numbers and impaired function of IL-10^+^ B cells [28], suggesting that the host may limit acute inflammatory responses through compensatory expansion of Bregs.

In addition to directly suppressing effector T cells, Bregs can maintain immune tolerance by regulating Tregs. Studies have found that B cells depend on GITR-GITRL signaling to maintain the number and function of Tregs: B-cell deficiency leads to a marked reduction in Tregs, whereas adoptive transfer of normal B cells restores this alteration [32]. Furthermore, B cells can induce CD4^+^CD25^−^ T cells to differentiate into two types of regulatory T cells, *Foxp3*^+^ and *Foxp3*^−^ [32], further supporting that Bregs participate in immune homeostasis through multiple mechanisms.

However, this protective effect is not sustained. In patients with dilated cardiomyopathy, the function of IL-10^+^ B cells is markedly reduced [18,53], suggesting an impaired regulatory B-cell response in chronic disease. However, whether this alteration directly contributes to persistent inflammation and adverse myocardial remodeling remains unclear.

### 4.3. B-Cell Functional Plasticity Provides New Targets for Precision Immunotherapy

In recent years, a growing body of evidence suggests that B-cell subsets are not fixed, but are continuously regulated by the local inflammatory microenvironment and exhibit pronounced functional plasticity. Therefore, the classification of B cells as pathogenic or protective may depend on disease etiology, stage, and local immune context, and the available evidence does not yet support a uniformly pathogenic or protective role for individual B-cell subsets. The cholinergic anti-inflammatory pathway is one of the important regulatory mechanisms. Preclinical studies in experimental viral myocarditis models have shown that the cholinesterase inhibitor pyridostigmine can improve ventricular remodeling and delay progression toward dilated cardiomyopathy through activation of α7nAChR on B cells [20]. Similarly, trehalose improves B-cell function through *AMPK/ULK1*-mediated autophagy and alleviates myocardial inflammation in experimental models [54]. These findings provide preclinical support for targeting B-cell functional pathways, but their therapeutic efficacy and safety in patients with myocarditis remain to be established.

## 5. Translational Value of B Cells in Myocarditis Clinical Management and Precision Regulation Strategies

With the deepening understanding of the immunopathogenesis of myocarditis, B cells have gradually evolved from traditional effector cells into important research targets spanning disease diagnosis, risk assessment, and therapeutic intervention (Figure 3). In recent years, extensive studies have shown that B-cell-related biomarkers, autoantibody profiles, and B-cell functional status can comprehensively reflect disease activity and immune response characteristics [55,56], providing new approaches for precise immune phenotyping and personalized therapy in myocarditis.

### 5.1. B-Cell-Related Biomarkers Facilitate Disease Classification and Risk Assessment

Autoantibodies have been investigated as potential clinical biomarkers of abnormal humoral immune activation in myocarditis. In patients with acute viral myocarditis and dilated cardiomyopathy, various autoantibodies, including anti-α-cardiac myosin (MyHC-α), anti-adenine nucleotide translocator (ANT), and anti-β1-adrenergic receptor antibodies, can be detected in serum, and their titers have been reported to be associated with the degree of myocardial inflammation, decline in left ventricular function, and NT-proBNP levels [18,22,24,45,46,53,57]. Furthermore, with disease progression, B-cell immune responses can gradually expand from viral antigens to cardiac self-antigens, namely epitope spreading, a process considered an important immunological event driving the evolution from acute myocarditis to dilated cardiomyopathy [16,17].

In addition to autoantibodies, B-cell functional status has also emerged as a new evaluation indicator in recent years. In experimental viral myocarditis models, B10 cells undergo transient compensatory expansion during the acute phase of viral myocarditis [21,28]. In human studies, however, the number and function of IL-10^+^ Bregs are markedly reduced in patients with dilated cardiomyopathy [18,32,48,53]. Meanwhile, TNF-α^+^ B cells persistently increase and are closely associated with myocardial fibrosis and deterioration of cardiac function [18,51]. These findings suggest that B-cell subset composition may have potential as a biomarker of immune status or disease progression; however, its diagnostic and prognostic utility requires validation in larger, prospective clinical cohorts.

In recent years, single-cell sequencing and B-cell receptor (BCR) repertoire analysis have further expanded the scope of B-cell biomarkers. Features such as BCR clonal expansion, biased V(D)J gene usage, and immunoglobulin class switching can dynamically reflect antigen-driven humoral immune responses [26,38]. These BCR repertoire features represent promising investigational biomarkers for immune phenotyping, although their clinical utility has not yet been established. In the future, integrating BCR repertoire features, autoantibody profiles, and B-cell subset information with clinical indicators may help define more precise immune phenotypes and generate testable biomarker candidates. Their value for early disease identification or risk stratification should be evaluated in adequately powered, prospective, and independently validated cohorts.

### 5.2. Targeting the B-Cell Immune Network Is a New Direction for Myocarditis Therapy

Clinical evidence for B-cell-targeted therapy in myocarditis remains limited. In patients with antineutrophil cytoplasmic antibody-associated vasculitis, including eosinophilic granulomatosis with polyangiitis complicated by myocarditis, rituximab treatment has been associated with improvement in cardiac function and reduction in inflammatory parameters [58,59]. However, these observations primarily derive from patients with systemic immune-mediated diseases with cardiac involvement rather than myocarditis-specific clinical trials, and therefore should not be directly extrapolated to establish the efficacy of rituximab in myocarditis. In ICI-associated myocarditis, persistently elevated B-cell chemokines such as *CXCL13* suggest that B cells participate in disease pathogenesis, and some patients with suboptimal responses to glucocorticoid therapy may also benefit from B-cell-targeted therapy [25,60,61].

However, a growing number of studies suggest that nonselective depletion of all B cells is not the optimal therapeutic strategy. Because Bregs play important roles in suppressing Th17 responses, maintaining Treg homeostasis, and limiting the spread of inflammation [20,52,62], comprehensive B-cell depletion may also impair their immune-protective functions. Accordingly, emerging therapeutic approaches are increasingly exploring B-cell functional reshaping, including selective inhibition of pathogenic B-cell pathways while preserving or enhancing immunoregulatory Breg subsets. However, most of these approaches remain preclinical or conceptual and require further validation in myocarditis.

### 5.3. Precision Regulation of B-Cell Subsets Is an Important Future Direction

Most current evidence for precision regulation of B-cell-associated pathways remains preclinical. In experimental myocarditis models, blockade of the Th17/IL-17-B-cell axis can inhibit B-cell proliferation and anti-ANT autoantibody production and alleviate myocardial inflammation [63,64]. The IL-23/IL-17 signaling pathway has also been considered an important therapeutic target in experimental autoimmune myocarditis [65]. On the other hand, promoting Breg expansion has become a new research focus. PGE_2_ can promote the expansion of IL-10^+^ B10 cells through MAPK/AKT-AP1 and AhR signaling [28], and trehalose can improve B-cell immunoregulatory function through AMPK/ULK1-mediated autophagy [54]. Furthermore, peptide vaccines designed on the basis of pathogenic B-cell epitopes and precision immunotherapy targeting specific BCR clones provide new ideas for future active immune intervention [66,67]. Whether these strategies can be translated into effective and safe treatments for patients with myocarditis remains unknown.

Notably, with the development of single-cell omics and BCR repertoire technologies, future B-cell therapeutic strategies will place greater emphasis on individualized precision intervention. By integrating pathogenic B-cell clone identification, dynamic BCR repertoire monitoring, and functional subset analysis, it may be possible to achieve precise immune phenotyping of patients across different etiologies and disease stages, thereby guiding treatment decisions, improving efficacy, and reducing immunosuppression-related adverse effects.

Several emerging strategies may represent potential therapeutic approaches for modulating B-cell-associated immune responses in myocarditis. Targeting the *BAFF*/*APRIL* axis may reduce B-cell survival and plasma-cell differentiation, while *BTK* inhibition may suppress BCR-dependent B-cell activation and downstream immune responses [68,69]. CAR-Treg cells may provide a novel approach to restore antigen-specific immune tolerance by combining engineered antigen recognition with the regulatory functions of Tregs [70]. In addition, nanoparticle-based delivery may facilitate targeted delivery of immunomodulatory agents to relevant immune cell populations [31]. However, these approaches have not yet been sufficiently validated as B-cell-directed therapies in myocarditis and should currently be considered potential or experimental strategies requiring further investigation.

### 5.4. Key Challenges in the Clinical Translation of B-Cell Research

Despite the continued progress of B-cell research, which has provided new directions for precision diagnosis and treatment of myocarditis, substantial challenges remain for widespread clinical application. First, existing studies generally have small sample sizes and are mainly limited to specific populations, including viral myocarditis, fulminant myocarditis, and ICI-associated myocarditis. The commonalities and differences in B-cell immune responses across different etiologies and disease stages have not been validated by large-scale, multicenter prospective studies [26,38,60]. Second, the reported autoantibody profiles and BCR repertoire features have not yet been standardized with respect to detection methods and evaluation systems, and their sensitivity, specificity, and reproducibility still require further validation in larger, prospective clinical studies [26,38]. In addition, most current studies rely mainly on peripheral blood samples, whereas myocardial B-cell infiltration, tissue-resident B cells, and their interaction with the local immune microenvironment remain insufficiently studied, limiting a comprehensive understanding of disease pathogenesis to some extent. Notably, there is currently no unified standard for defining pathogenic B cells, and their phenotype, antigen specificity, and functional status are still poorly characterized. Likewise, specific molecular markers that can reliably identify protective Bregs are lacking. Therefore, the key to future precision immunotherapy lies not only in discovering new biomarkers, but also in establishing a functional classification system for pathogenic B cells and immunoregulatory B cells, so as to achieve precise intervention of different B-cell subsets rather than nonselective depletion of all B cells.

## 6. Conclusions and Future Perspectives

In recent years, advances in single-cell sequencing, BCR repertoire sequencing, and multi-omics technologies have expanded the understanding of B cells in myocarditis from a traditional source of autoantibodies to important regulators of immune responses and disease outcomes. Existing evidence indicates that B cells are associated with the initiation, persistence, and myocardial remodeling of myocarditis through BCR clonal expansion, autoantibody production, and dynamic reshaping of functional subsets, and that functional imbalance of these subsets may be an important mechanism driving disease chronification. However, the specific markers, antigen specificity, and dynamic evolution patterns of pathogenic B cells remain unclear. Moreover, the clinical utility of BCR repertoires and autoantibody profiles as biomarkers remains to be established, as standardized assays and adequate validation of their sensitivity, specificity, and reproducibility are currently lacking. Their potential value in disease classification, risk stratification, and therapeutic response assessment therefore requires validation in large-scale, prospective, and independently validated studies. In the future, integrating emerging technologies such as single-cell omics, spatial transcriptomics, and BCR repertoire sequencing may help systematically characterize B-cell immune heterogeneity and its regulatory networks and generate testable biomarker candidates and therapeutic hypotheses. Whether these findings can ultimately support clinically useful immune phenotyping or personalized treatment remains to be determined.

## Figures and Tables

**Figure 1:**
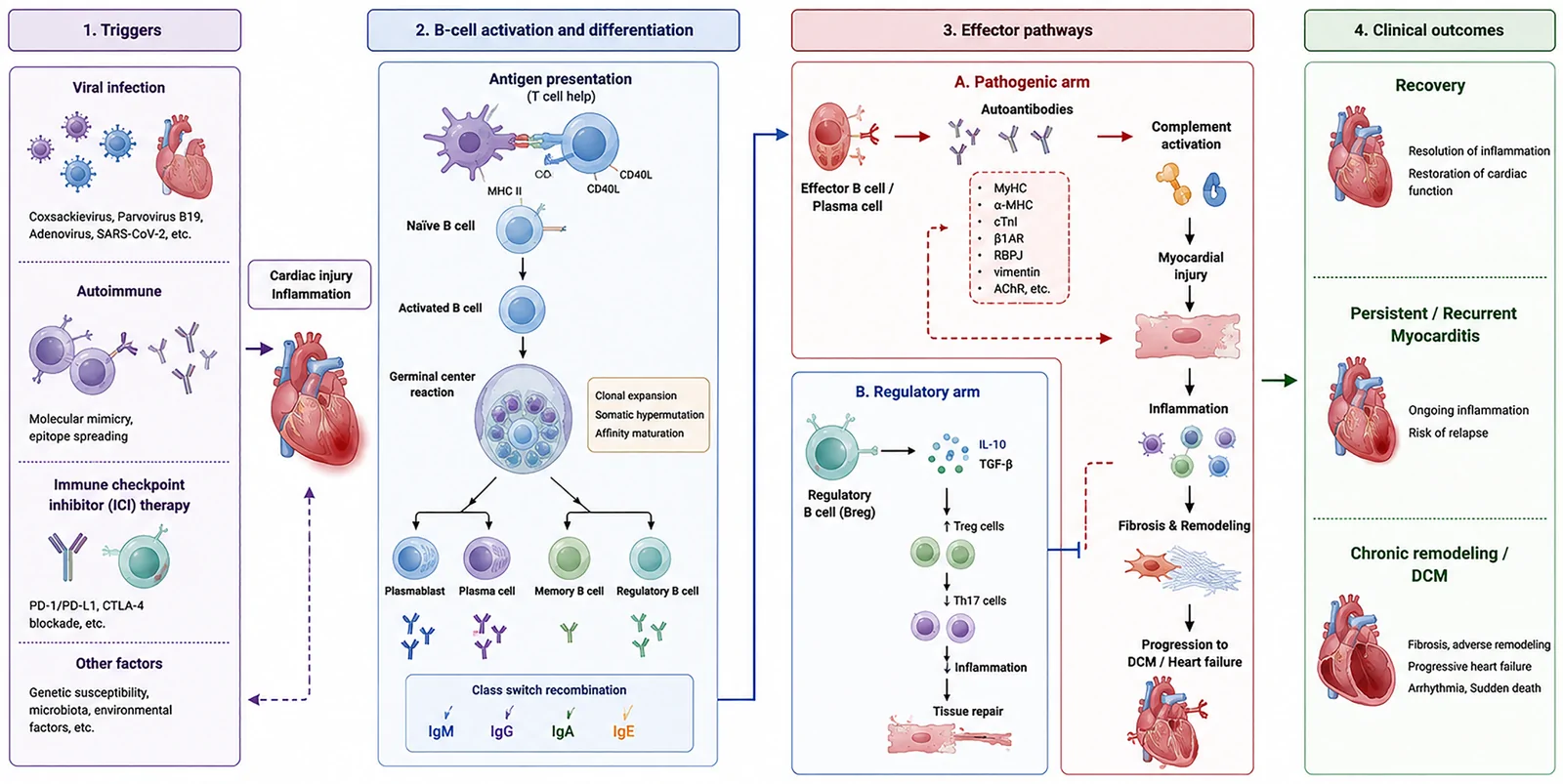
Schematic overview of B-cell-mediated mechanisms in myocarditis.

**Figure 2:**
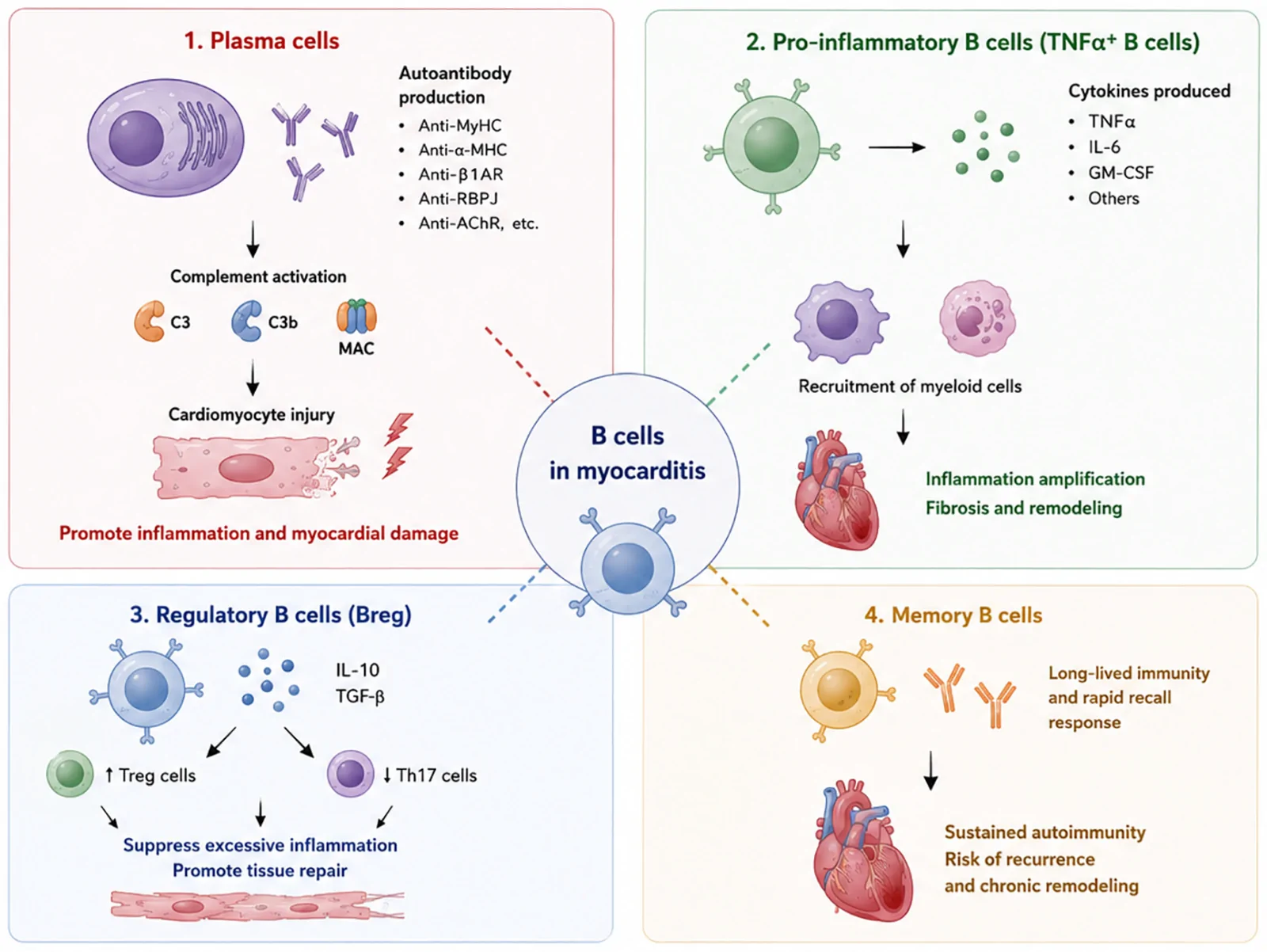
Distinct roles of B-cell subsets in myocarditis.

**Figure 3:**
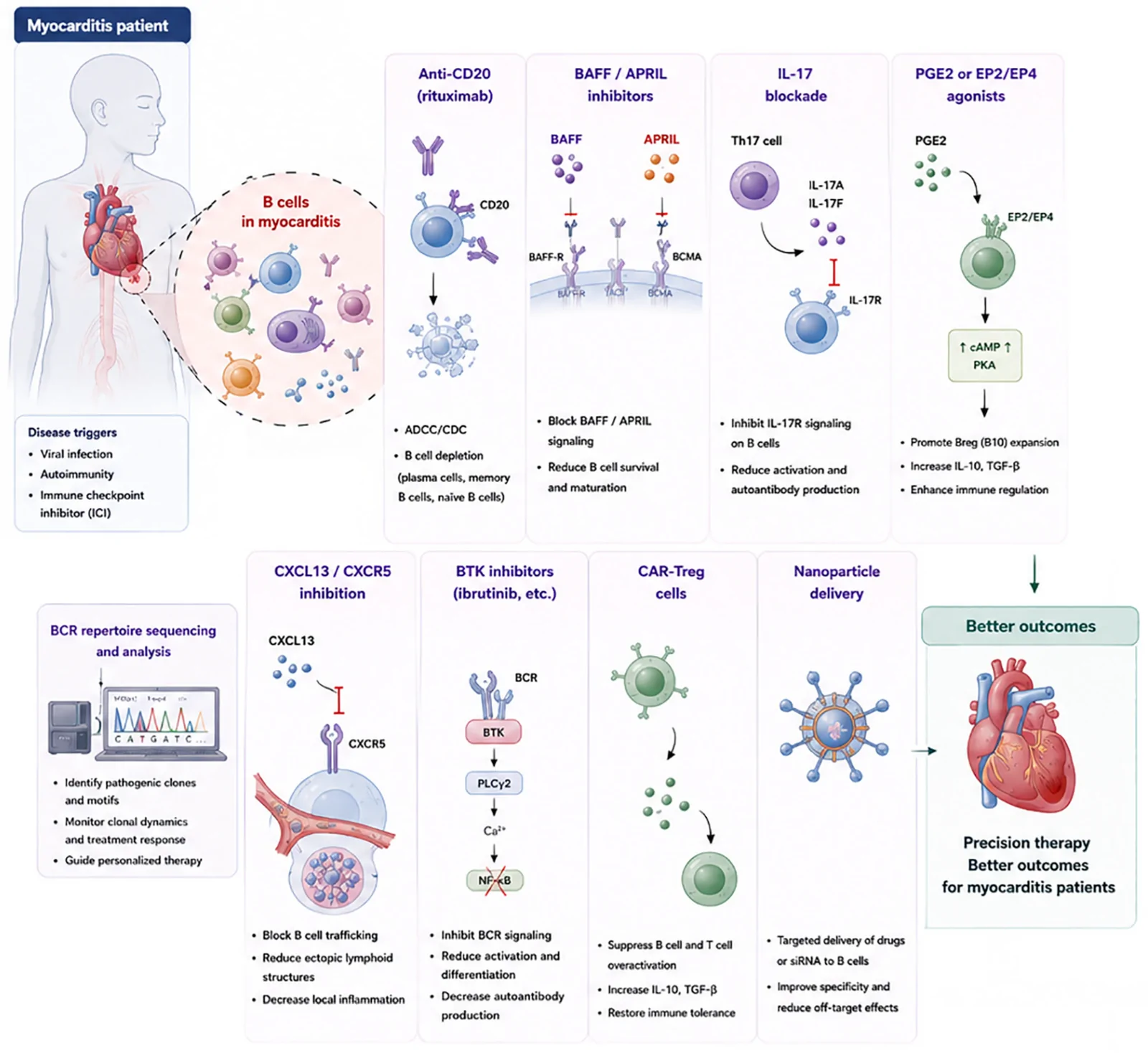
Therapeutic approaches directed against B cells and their effector pathways in myocarditis.

## Data Availability

No new data were created or analyzed in this study. Data sharing is not applicable to this article.
